# Impact of climate warming on the foraging behavior of northernmost distributed primates

**DOI:** 10.1038/s41598-025-09308-0

**Published:** 2025-07-25

**Authors:** Ema Nagahara, Ayaka Tsuchihashi, Takumi Yoshida, Kosuke Hayashi, Genki Yamada, Takayuki Ogura, Mone Ito, Hirokazu Kurihara, Koji Tojo, Takuya Matsumoto, Masaki Takenaka

**Affiliations:** 1 Department of Biology, Faculty of Science, Shinshu University, Asahi 3-1-1, Matsumoto, 390-8621 Japan; 2 Graduate School of Science and Technology, Shinshu University, Asahi 3-1-1, Matsumoto, 390-8621 Japan; 3 Graduate School of Medicine, Science and Technology, Shinshu University, Asahi 3-1-1, Matsumoto, 390-8621 Japan; 4 NHK Enterprises, Inc., 150-0047 Kamiyama 4-14, Shibuya, Tokyo Japan; 5 G-Vision, Inc., 182-0006 Nishitsutsujigaoka 1-54-12, Chofu, Tokyo Japan; 6 Kozo Production, 150-0042 Udagawa 37-10-301, Shibuya, Tokyo Japan; 7 Vision1 Inc., 150-0047 Kamiyama 10-7, Shibuya, Tokyo Japan; 8 Institute of Mountain Science, Shinshu University, Asahi 3-1-1, Matsumoto, Nagano 390-8621 Japan; 9 Institute of Life and Environmental Sciences, University of Tsukuba, Tennodai 1-1-1, Tsukuba, Ibaraki 305-8577 Japan; 10 Mountain Science Center, University of Tsukuba, Tennodai 1-1-1, Tsukuba, Ibaraki 305-8577 Japan

**Keywords:** DNA metabarcoding, Dietary plasticity, Global warming, Japanese macaques, Mountain ecology, Primates, Climate-change impacts, Animal behaviour

## Abstract

Climate warming has profound effects on animal behavior, ecology, as well as on human activities. As the number of extreme weather events increases with climate warming, organisms are facing unprecedented environmental challenges. In temperate regions, winter food scarcity poses a critical survival challenge for many animals. This study investigated the unique foraging behavior of the Kamikochi population of Japanese macaque, *Macaca fuscata*, which forage for aquatic insects during severe winters with scarce food availability. In February 2024, a thermal event with rising temperatures and rain triggered snowmelt and river flooding, which made it difficult for the macaques to access aquatic food. Comparisons of foraging behavior during periods with and without the thermal event showed that Kamikochi macaques reduced their foraging on aquatic insects inhabiting streams during the thermal event. These findings highlight the ecological impacts of heating events expected to become more frequent due to climate change. Japanese macaques in Kamikochi provide valuable insights into how wildlife behavior may change in response to these events.

**Supplementary Information:**

The online version contains supplementary material available at 10.1038/s41598-025-09308-0.

## Introduction

Rapid climate warming is forcing many organisms to deal with extreme heat and rapid temperature rises, presenting novel and potentially lethal conditions they have not encountered before^1,2^. Extreme weather events associated with climate warming have been reported to cause significant damage to ecosystems^2,3^, result in mass mortality events^4,5^, lead to distribution changes^6^, and changing the timing of breeding and phenology^7,8^. In particular, vulnerable mountain ecosystems are strongly impacted by rising temperatures and humidity changes, affecting vegetation dynamics and animal ecology^9,10^. It is predicted that the increasing likelihood of even thermal events in the future will further impact these ecosystems^3,5,11^. However, it generally remains unclear how climate change affects the ecology and behavior of organisms^2,3,6^.

In assessing the future threat of warming to land vertebrates, it has been reported that mid-latitude regions are the most exposed to extreme events^2,12^. Additionally, small islands, coastal regions, and high mountain ranges are also noted as being the most severely affected^13^. In particular, alpine regions are more vulnerable to warming compared to other ecosystems^14,15^. However, there is little direct evidence regarding climate warming in alpine regions. Furthermore, considering snow-covered mountains, it has been reported that the upward migration of the snowline and tree line due to warming significantly increases the absorption of solar radiation, which in turn accelerates the warming process^15^. The high mountain ecosystems of the Japanese Islands, which are relatively small islands, are sensitive to the above changes, and are therefore a suitable area to investigate the impacts of climate warming.

**Fig. 1:**
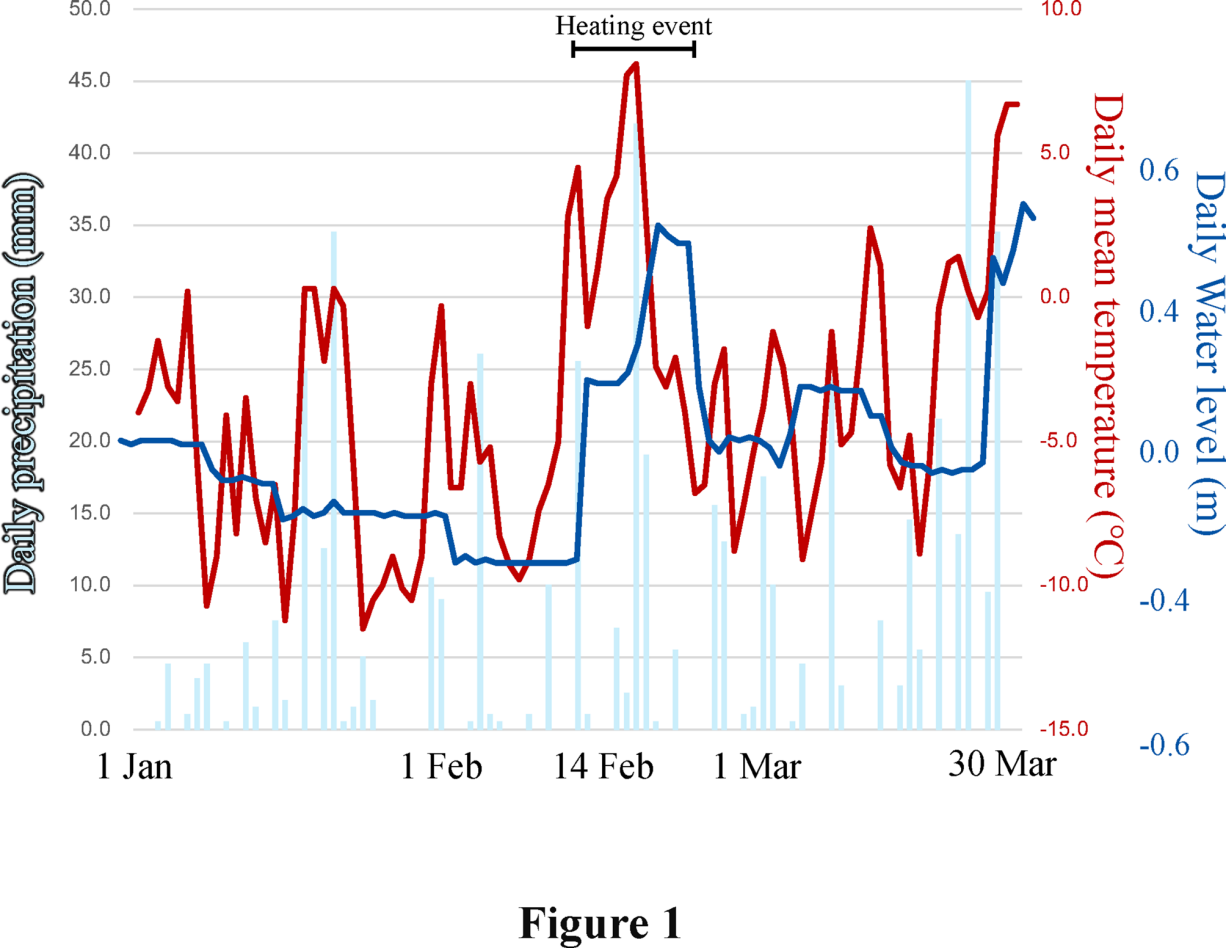
Daily mean temperature and precipitation in Kamikochi and changes in the stream water level of the Azusa River in 2024.

In temperate regions, winter food scarcity places many animals at risk of mortality^16–18^. Additionally, in cold, high-altitude regions with heavy snow, the ground is completely covered with snow, making access to food difficult. Foraging strategies become a crucial factor to enable survival in such areas. Among primates, which are primarily distributed in tropical regions^19^, the Japanese macaque, *Macaca fuscata*, distributes within the northernmost latitude of any non-human primate^19,20^. In Japanese macaques, the Kamikochi population (Kamikochi macaques) inhabits the coldest regions^21,22^ (Fig. 1, S1). The Kamikochi macaques are a rare primate species that overwinter in a snow-covered area during the harsh winter season and even climb to alpine areas in the summer^22,23^. In addition, the Kamikochi macaques demonstrate unique foraging behaviors in winter, such as foraging aquatic organisms in streams^21,22^. Kamikochi has many springs and small tributary streams (Fig. S2). The main stream, the Azusa River, has a low water level, allowing frequent access to bodies of water where Kamikochi macaques can forage aquatic organisms, even eating fish^21,22^ . As far as we know based on previous studies, no primate that captures and forages the larvae of aquatic insects from streams other than the Kamikochi macaques have been reported. In this study, we confirmed frequent foraging behaviors targeting aquatic insects in water habitats through DNA metabarcoding analysis and direct observation using a high-resolution camera.

Also, rather than using simulated data, we obtained direct data on changes in animal behavior and ecology due to climate warming. The findings provide insights into how animals respond to environmental changes caused by climate warming, and it may help not only to clarify changes in animal behaviors related to past climate shifts but also their responses to future climate changes. In mid-February 2024, the coldest period of the year. A high-temperature event with mean temperatures above 0°C occurred simultaneously with a precipitation event, resulting in snowmelt and leading to stream flooding (Fig. 1). We defined the high-temperature event as the period from 14 February 2024, when the average temperature rose sharply, to 28 February 2024, when the temperature dropped sharply (Fig. 1). This event is likely to occur frequently as climate warming progresses. To investigate how the foraging behavior of Kamikochi macaques changed before and after this snowmelt event, we analyzed their foraging behavior using both direct observation data and DNA metabarcoding analysis of feces.

## Results and discussion

### Insect foraging behavior in streams

**Fig. 2:**
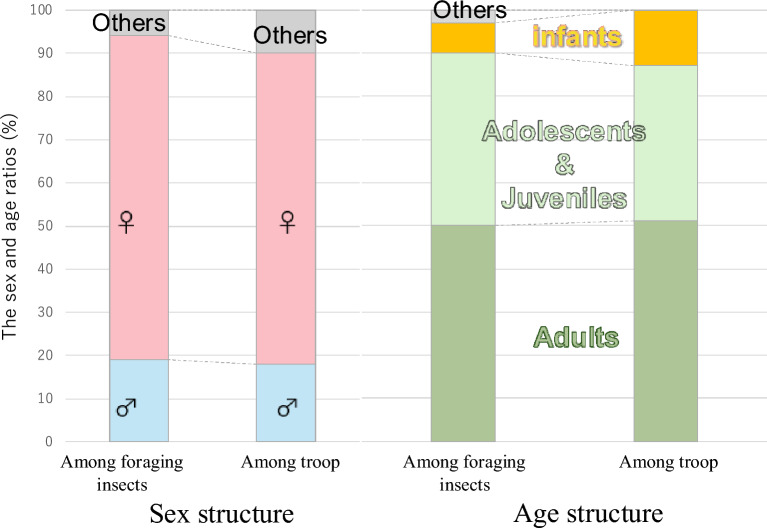
Sex and age ratios (infants, juveniles, adolescents, and adults) of Kamikochi macaques foraging on aquatic insects were compared with those of the overall Kamikochi troop.

We observed the behavior of three troops (dubbed the KT, KK, and KM troops; Fig. S3). On almost all survey days (27 out of 33 days), foraging behavior was observed in water habitats based on the direct observation (main stream, 53.7%; small tributary streams, 56.1%; wetlands, 24.4%; pond, 2.4%; Table S1, Fig. S2). Also, on days when the Kamikochi macaques did not go to water habitats in our observation, heavy snow occurred throughout the day, and the Kamikochi macaques moved very little. Direct observations did not capture any Japanese macaques on days with heavy snowfall, and our direct observations similarly indicated that the macaques exhibited minimal activity under such conditions. It has been also reported that in snowy regions, the movement range of the Kamikochi macaques decreases on heavy snow days^18,20^. Following a day of heavy snow, Kamikochi macaques were observed foraging in water habitats for extended periods, suggesting that water habitats play an important role as foraging locations during the winter. After analyzing the foraging behavior of Kamikochi macaques in water habitats using 253.8 min of camera-based observations, no significant differences were found in the age or sex ratios of the Kamikochi macaques foraging on aquatic insects (Fig. 2). Even infants (zero-year-old) were observed foraging, suggesting that aquatic insects may serve as an important food source in winter in Kamikochi.

Primates forage replacement foods (fallback foods) when high-quality food is scarce^24–26^. The forage of replacement foods has been reported to differ by sex and age^27^. For example, larger bodied adults often consume mature leaves, which are readily available but fibrous and difficult to digest, whereas smaller juveniles tend to forage insects, which are higher in quality but less abundant^28–30^. Due to differences in digestive ability by age among Japanese macaques, mature leaves are considered difficult for juveniles to consume^30^. This tendency for food use to differ based on body size differences such as age and sex is known as the Jarman–Bell principle, which states that smaller animals consume higher quality foods than larger animals^31^.

**Fig. 3:**
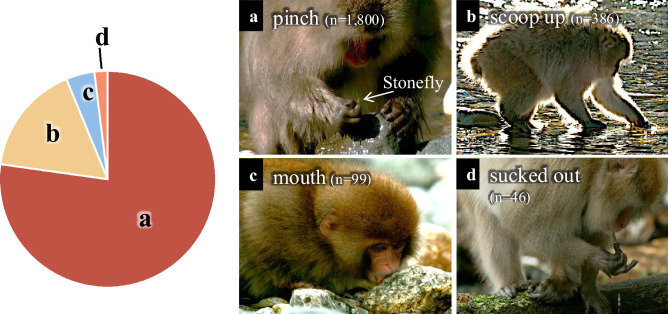
Proportions of the foraging methods used by the Kamikochi macaques to forage for aquatic insects were categorized as: (**a**) pinch, pinched insects using their fingers, (**b**) scoop up, scooped up insects with their hands, (**c**) mouth, foraged directly with their mouths and (**d**) sucked out, sucked out insects from insect nests.

In Kamikochi during winter, plant-based food resources, which are the primary diet, become scarce. Spring-fed streams remain unfrozen and continue to flow, functioning as valuable foraging sites^32^. Therefore, even in winter, access to rivers is easy, and by turning over stones, relatively high-quality aquatic insects can be found. In the case of Kamikochi macaques, no significant differences were detected in the sex or age ratios of individuals foraging aquatic insects. Insects can be advantageous in terms of calories when consumed in large quantities^33^. Because they are easy to obtain, it is believed that they are foraged regardless of age or sex. Also, the most frequently detected behavior involved Kamikochi macaques turning over stones in streams, and pinching aquatic insects found under the stones such as crawlers like Perlodidae (Plecoptera) or swimmers like *Ameletus* sp. (Ephemeroptera), using their fingers (77.2%; Fig. 3; Supplementary Information 1). Also, some these crawler and swimmer species that had fallen off stones and were carried away by water flow were frequently scooped up by Kamikochi macaques (16.6%; Fig. 3; Supplementary Information 2). Additionally, tube case-making groups such as *Eubasilissa regina* (Trichoptera) were sucked out (Fig. 3; Supplementary Information 3), and for hard nests that could not be peeled off from rocks with their fingers, such as those of Trichoptera, they foraged directly with their mouths (Fig. 3; Supplementary Information 4). Kamikochi macaques exhibited flexible foraging strategies in response to conditions of aquatic insects. It can be inferred that aquatic insects, which can be stably foraged during periods of food scarcity in winter, are an important dietary item for Kamikochi macaques. Although not the focus of this study, behaviors such as foraging aquatic plants were also frequently observed in these small tributary streams (Supplementary Information 5).

### DNA metabarcoding

The camera-based observations are limited to information that could be visually confirmed. Therefore, DNA metabarcoding analysis was conducted using fecal samples of Kamikochi macaques (n = 175). As a result, more insect species were detected than in previous studies^22^ or from our direct observations (Table 1, S2, S3). As for species that had not been observed directly, terrestrial insects were also detected, indicating that the Kamikochi macaques consumed a greater variety of insect species than initially thought. Although insects are much smaller in body size compared to Japanese macaques, their biomass within river ecosystems is substantial, and they are relatively easy to collect. In Kamikochi during winter, streams fed by abundant spring water that do not freeze are particularly valuable as food resources^32^**.** It has been suggested that primates tend to consume small insects only when they are available in large quantities^25^. Since aquatic insects in river ecosystems can be easily collected in large numbers, they likely play an important role as replacement food resources during winter^34,35^. Regarding terrestrial insects, Kamikochi macaques are reported to frequently forage tree bark in winter^36^, suggesting that they may also forage for insects under the bark. In previous studies using molecular markers, it was reported that insects were not directly foraged , but were instead indirectly foraged when foraging fruits or plants that contained insects^35,37^. Since most primates are diurnal, dietary analysis using fecal DNA samples has rarely been conducted. However, this can be considered a powerful tool for uncovering previously unknown aspects of their diets^21,35,38^.

**Table 1:** List of insect groups detected using DNA metabarcoding analysis of feces from Japanese Kamikochi macaques.

Order	Family	Main habitat	Number of detected species in Kamikochi	Number of detected species per troop (normal time) *			Number of detected species per troop (during the heating event) **		
				KT	KK	KM	KT	KK	KM +
Ephemeroptera	Ameletidae	Stream	2	1	0	2	0	0	–
	Ephemerellidae	Stream	1	1	0	1	0	0	–
	Leptophlebiidae	Stream	1	0	0	0	0	1	–
	Isonychiidae	Stream	1	1	1	0	0	0	–
Plecoptera	Perlodidae	Stream	2	2	2	1	0	0	–
	Nemouridae	Wetland	1	1	1	0	0	0	–
Hemiptera	Miridae	Land	4	3	0	2	1	0	–
	Cicadellidae	Land	1	0	0	1	0	0	–
	Aphididae	Land	2	2	0	0	0	0	–
Psocodea	Psocidae	Land	2	2	0	0	1	0	–
	Caeciliusidae	Land	2	2	0	0	0	0	–
	Stenopsocidae	Land	1	1	0	1	0	0	–
Coleoptera	Cerambycidae	Land	1	0	0	0	0	1	–
	Erotylidae	Land	1	0	0	1	0	0	–
Diptera	Cecidomyiidae	Land	4	2	1	3	0	0	–
	Tipulidae	Land	1	1	0	0	0	0	–
	Lauxaniidae	Land	1	0	0	1	0	0	–
Lepidoptera	Noctuidae	Land	1	1	0	0	0	0	–
	Pieridae	Land	4	3	0	1	1	1	–
	Zygaenidae	Land	2	0	1	0	0	1	–
	Tortricidae	Land	1	1	0	0	0	0	–
	Unknown	Land	1	0	0	1	1	0	–
Trichoptera	Hydropsychidae	Stream	1	1	0	0	0	0	–
	Limnephilidae	Stream	1	1	0	0	0	0	–
	Phryganeidae	Wetland	2	2	2	0	1	0	–

*, The survey period excluding the dates of the heating event; **, The “heating event” was defined as a period during which the average temperature was above 0 °C, precipitation occurred, and river water levels rose, + , No data because no fecal samples were collected from the KM troop during the heating event.

Among primates worldwide, many species forage for insects^34,37,39^. However, accessing streams to forage for aquatic insect larvae is a unique behavior observed exclusively in Kamikochi, as most primates exhibit aquaphobia. In a study by Mallott et al.^38^ using high-throughput sequencing of fecal DNA, foraging for aquatic groups, Ephemeroptera and Chironomidae (Diptera), was reported. However, since the habitat of the Saddleback Tamarins is high in the trees and the study period coincided with the adult emergence season, it is likely that the foraged insects were in their adult stage, which inhabits land. Although many researchers have investigated the dietary items of Japanese macaques in various regions, aquatic insects have not been reported outside of Kamikochi^21,40–42^.

### Climate warming

Mammals require additional food to maintain body temperature, and they cannot sustain this under cold conditions with limited food availability^43^. In fact, many primates are sparsely distributed in temperate regions with harsh winters^19^. The foraging behavior of Kamikochi macaques on aquatic insects is a novel behavior that has not been observed in other regions. The novel foraging behavior in Kamikochi macaques during the winter has not only enabled them to survive the harsh winter, but might also help them secure a more stable food source. The novel foraging behavior in Kamikochi macaques during the winter has not only enabled them to survive the harsh winter, but might also help them secure a more stable food source.

**Fig. 4:**
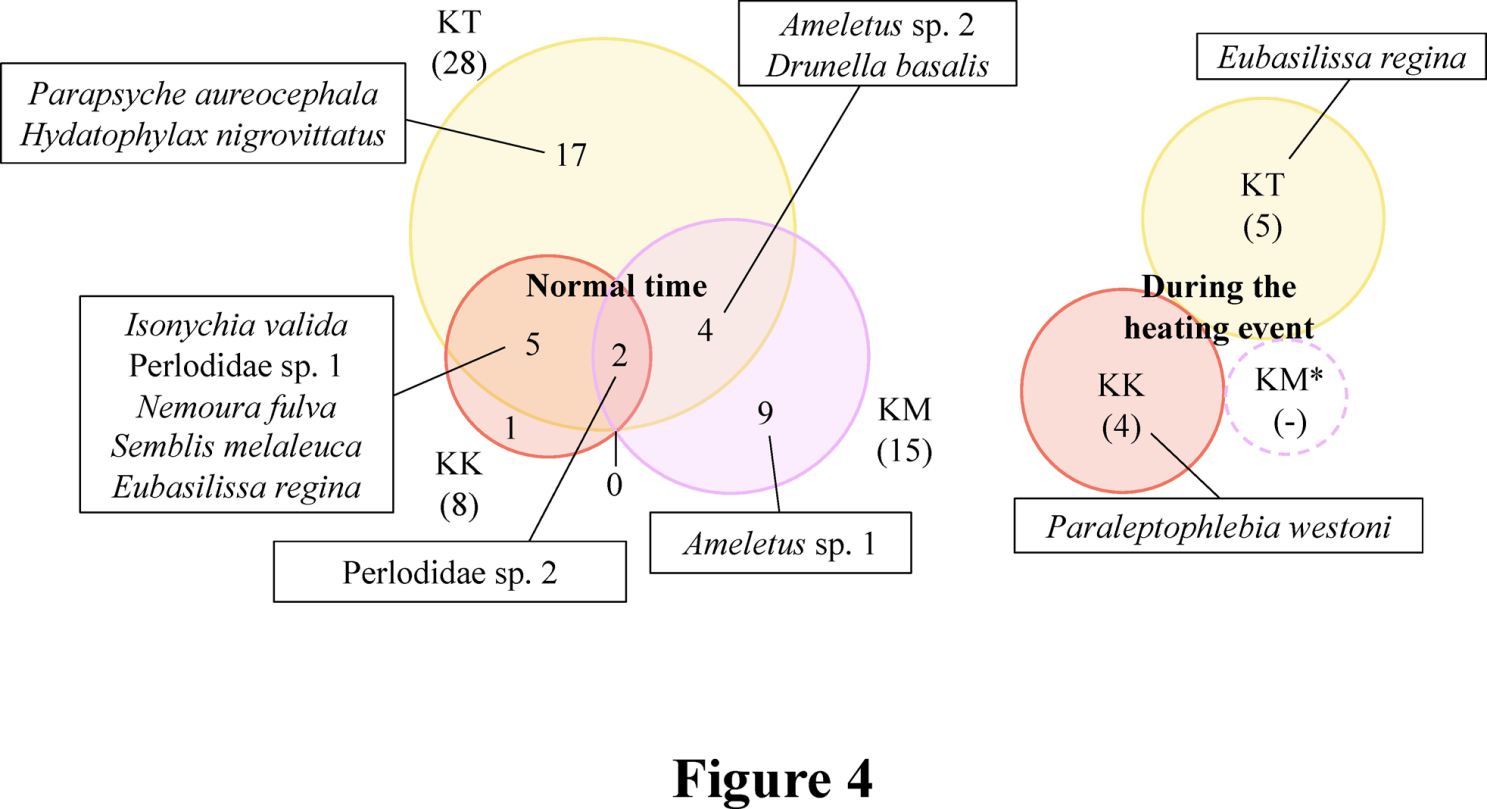
Insect species detected through DNA metabarcoding analysis based on the mtDNA 16S rRNA region using the feces of Kamikochi macaques are shown by troop (see Table 1 for details). The size of the circles reflects the number of species detected, with the numbers inside each circle indicating the number of species detected. The numbers in parentheses next to the troop names indicate the total number of species detected in each troop. The results between normal time and the thermal event were compared. Among the detected species, aquatic insects are shown.

Easy access to water bodies, facilitated by lower water levels in the main stream due to snowfall and snow accumulation during the winter^24^, is an important factor in this process. Thermal (high-temperature) events in mid-February 2024 led to an increase in stream water levels (Fig. 1), reducing the Kamikochi macaques’ access to the main stream (Table S1). The relationship between their novel foraging behavior and thermal events is well-suited to investigate how warming impacts the foraging behavior and ecology of mammals. We compared the insect species foraged by the troops between normal time and the thermal event (Table 1) . Based on the results of DNA metabarcoding analysis, diring a high-temperature event , the proportion of aquatic insects among all foraged insects decreased: KT, 46.2% → 28.6%; KK, 71.4% → 40.0%; KM, 30.8% → 0% (Table S2). Also, analysis of the list of foraged insect species revealed different community structures before and after the thermal event (Fig. 4, S4-S6).

In mid-February, temperatures remained consistently above 0°C, accompanied by rain (Fig. 1). Under such climatic conditions of temperature rise and rain, "Rain on Snow (ROS)" events are reported to cause snowmelt^44,45^. ROS impacts the hydrological cycle by accelerating the melting of snow^44^. In field observations, as the snow melted and the ground became exposed in many areas, the frequency of foraging on land increased. Additionally, snowmelt caused an increase in the water level of the main stream (Table S1). An increase in water level was also detected at a gauge point located approximately 20 km downstream from Kamikochi (Fig. 1).

Various scenarios have been proposed in accordance with greenhouse gas emissions, but in all of these scenarios, many land vertebrate species will experience extreme thermal events^2^. Also, it is estimated that extreme thermal events will increase in the future^2,3,5,11^. From a long-term perspective, with the frequent occurrence of high temperatures, an increase in precipitation as rain rather than snow will lower the albedo due to the presence of wet snow and subsequent refreezing, thereby accelerating further snowmelt^44^. In other words, the frequent increases in stream water levels may result in a loss of stable access to aquatic organisms. Similar to other regions, the absence of stable food sources could lead to a shift in diet towards tree bark and winter buds, causing a change in the foraging behavior of Kamikochi macaques. Previous studies simulating climate warming and examining the impacts of specific high-temperature events have predicted mass die-offs of organisms and ecosystem collapse^4,5,7^. However, this study is important in that it demonstrates, based on actual observations, that the behavior of mammals, specifically Japanese macaques, changes under high-temperature conditions.

Finally, the Kamikochi population of Japanese macaques inhabits the coldest regions of all non-human primates. Under such harsh conditions, a combination of environmental factors such as winter food shortages, low stream water levels, and flat geology has led to unique aquatic insect foraging behaviors. However, heating events caused have negatively impacted these behaviors. These heating events were predicted become more frequent worldwide as a result of global warming^3,5,11^. Our findings offer valuable insights into how animals respond behaviorally to these events, providing important information that can help in understanding the ongoing effects of heating worldwide.

## Methods

Observations of foraging behavior in primates have primarily been based on direct observations, but it is not always feasible to observe them continuously^46^. On the other hand, analyses using DNA does not involve direct observation of specimens, making it difficult to ascertain the condition or stage of insects, among other factors, resulting in limited information. By combining these two methods, we comprehensively analyzed the foraging behavior of Japanese macaques.

In this study, we investigated three troops of Japanese macaques inhabiting Kamikochi, each with different home ranges^22^. Direct observations were conducted by tracking each macaque troop in Kamikochi to observe their behavior. These observations were carried out over a 33-day period from January 12 to March 1, 2024. In addition, ad libitum sampling using high-end 4K cameras and handy cameras was conducted from 21 January 2022 to 8 February 2022 and 11 January 2023 to 22 March 2023. To supplement this, camera trap footage was analyzed to investigate foraging behavior. These traps were set in the same sites as previous studies and operated from January 19 to February 16, 2023. Notably, the camera trap data were available only for the KT troop in this study. To perform DNA metabarcoding analysis, fecal samples from Japanese macaques were collected. Sampling periods were as follows: KT troop from January 14 to February 28, 2024, KK troop from January 12 to February 28, 2024, and KM troop from January 23 to March 1, 2024. Troop identification was based on individuals that had been previously identified. For both DNA metabarcoding and home range tracking, analyses were conducted separately for each troop to demonstrate that the observed behaviors were not specific to a single troop. In contrast, video-based analyses were treated as a single dataset, as troop identity could not always be determined from the footage.

The temperature and precipitation data were obtained using observation data from the Research Center for Mountain Environment, Shinshu University (data collected at 1-h intervals) (http://ims.shinshu-u.ac.jp/~metims_web/index.php?sokuhou) and from the Japan Meteorological Agency (data collected at 1-h intervals). The changes in water level of the Azusa River flowing through Kamikochi were measured at Yoko Bridge (Figs. S3, S7), upstream of the target area, using an ultrasonic water level gauge, with data corrected for water temperature. However, there is a large amount of missing data because measurements could not be made during the winter. Therefore, the records from Shimojima Bridge, approximately 20 km downstream from Kamikochi on the Azusa River, were used instead (Fig. S7).

### Behavior observation by direct observation and video analysis

We studied three troops of Japanese macaques (KT, KK, and KM troops from south to north; Fig. S3) inhabiting the Azusa River in Kamikochi (Chubu Sangaku National Park, 36.25272, 137.66731). Direct observations were conducted from 12 January 2024 to 1 March 2024, according to Altmann^47^. Using data from infrared sensor cameras in a previous study^22^, as well as high-end 4K cameras [a 4K 2/3-type 3-chip CMOS shoulder-mount camcorder (PXW-Z750, SONY; ), RED DIGITAL CINEMA DSMC2 (HELIUM 8K S35, RED), a 4K expert handy camera (FDR-AX100, SONY), a Phantom Flex4K digital cinema camera (Flex4K, PHANTOM)], and a handy camera (GoPro 9, GoPro Inc.), we analyzed the foraging behavior for aquatic invertebrates of Kamikochi macaques from 21 January 2022 to 8 February 2022 and 11 January 2023 to 22 March 2023. High-end 4K cameras opportunistically captured the Japanese macaques’ behavior along the riverbanks. Regarding the frequency of use for each water body, we only used data from all cameras that clearly showed foraging for aquatic insects. In addition, during direct observations, we visually confirmed and recorded the types of water bodies where aquatic insect foraging occurred. We analyzed the sex, age based on the all came-based data, and foraging methods, and insect species foraged based on a high-resolution camera by the Kamikochi macaques based on 209 video recordings (totaling 253.8 min). In this study, troop identification was conducted by confirming the presence of multiple individually recognized macaques.

Since most of the cameras were set to capture the Japanese macaques in water habitats via ad libitum sampling, we extracted each foraging behavior individually. In this analysis, the three troops were treated in the same way. The sex of the Japanese macaques (only adults) was determined as follows: individuals with visible testicles were classified as males, individuals without visible testicles or with nipple elongation were classified as females, and the rest were classified as unknown. The ages of the Japanese macaques were categorized as “adults”, “adolescents”, “juveniles”, and “infants” (definition of age categories: Supplementary Information 6). The obtained sex and age structures of individuals that foraged aquatic insects were compared with those of all Kamikochi macaques (Tsuchihashi et al. 2025). Statistical analysis (Pearson’s Chi-squared test: X-squared = 6, df = 4, p-value = 0.1991) using R version 4.3.2^48^ revealed no significant differences (p > 0.05).

The foraging methods were categorized as “pinch”, “scoop up”, “suck out”, and "forage directly with mouth" (definitions of foraging methods: Supplementary Information 7).

### DNA metabarcoding

In a previous study using DNA metabarcoding analysis based on the mtDNA COI region^21^, several species of aquatic insects were detected, and numerous species that were not detected through direct observation were identified^22^. This issue arises from general insect DNA barcoding methods based on the mtDNA COI region, which fail to detect about half of the species present^49,50^. Therefore, we established a DNA metabarcoding method based on the mtDNA 16S rRNA region^50^, which has shown higher sensitivity for detection than general metabarcoding methods, and used this approach in the present study. The Japanese macaques in Kamikochi spend the night in the trees, so in the morning, feces that had fallen under the trees where they slept were collected. From 12 January 2024 to 1 March 2024, we ensured that we did not collect multiple fecal samples from the same individual by collecting only one sample from each tree (totally 175 fecal samples, 3 troops, 32 days). Additionally, we recorded the behavior of the troops the day before the feces were collected. The collected fecal samples were stored at -20°C.

The DNA analysis method and data analysis are shown in Supplementary Information 8. All raw sequencing data were registered with the NCBI (Accession number: SRX28187138- SRX28187169, Bioproject: PRJDB15936). The OUTs identified as Insecta through a BLAST search were subjected to NJ tree construction (1000 bootstrap) using MEGA ver. 7.0.26^51^. Using the obtained phylogenetic tree, a species delimitation analysis (genetic species estimation) was conducted, and for species that could not be identified through BLAST, species identification was performed based on phylogenetic data (Fig. S8; detail in Supplementary Information 9). For species molecular identification, we adopted a method that incorporates not only the BLAST percentage identity, but also phylogenetic interpretation. Using the detected species data, we performed a community analysis of the insect fauna consumed by the Japanese macaque groups. The read counts of detected sequences for each day were converted into a 0/1 dataset. This dataset was then transformed into the Jaccard dissimilarity index and analyzed using Ward’s method for clustering, followed by plotting, with R version 4.3.2^48^. We described the major habitats of aquatic insect species foraged by Japanese macaques according to Kawai and Tanida^52^.

## Supplementary Information

Below is the link to the electronic supplementary material.

Supplementary Information.

Supplementary Information.

Supplementary Information.

Supplementary Information.

Supplementary Information.

Supplementary Information.

Supplementary Information.

Supplementary Information.

## Data Availability

All methods information included in this manuscript. All raw sequencing data of metabarcoding were registered with the NCBI (Accession number: SRX28187138-SRX28187169, Bioproject: PRJDB15936; https://www.ncbi.nlm.nih.gov/sra/PRJNA1243855). To enhance the DNA reference database of insects in Kamikochi, all raw sequencing data of direct sequences obtained from insect tissues have been deposited in the DNA Data Bank of Japan (accession numbers LC859418–LC859545).
