# Supplementary material for: Impact of climate warming on the foraging behavior of northernmost distributed primates
Source: Sci Rep. 2025 Jul 25;15:27096. doi: 10.1038/s41598-025-09308-0 (PMC12297154; doi:10.1038/s41598-025-09308-0)
Supplement: Supplementary file 1 — Supplementary Information. [file 41598_2025_9308_MOESM1_ESM.pdf]

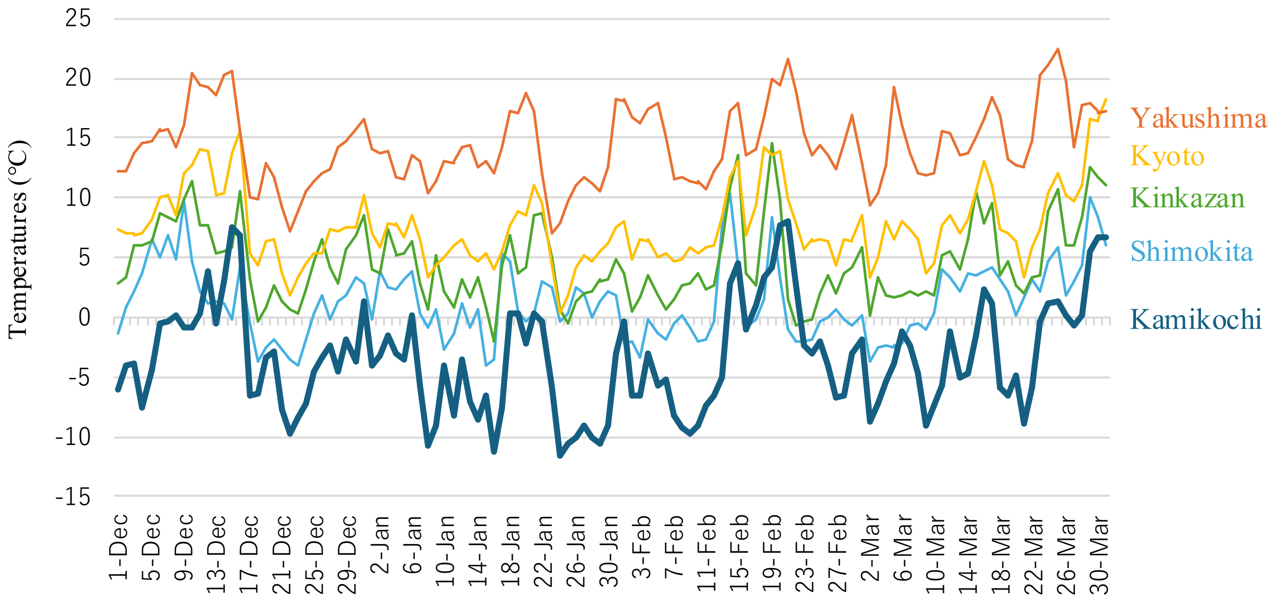

Figure S1 Daily mean temperatures in winter across various locations on the Japanese Islands where macaques inhabit, including Yakushima (southernmost area), Kyoto, Kamikochi (the study area), Kinkasan (island in northeastern Japanese Islands), and the Shimokita Peninsula (northernmost area) (Dec 1, 2023 – Mar 31 2024). The temperature in Kamikochi is lower than in any other region where macaques are distributed. In particular, it is colder than the Shimokita Peninsula, which is the northernmost distribution range of non-human primates.

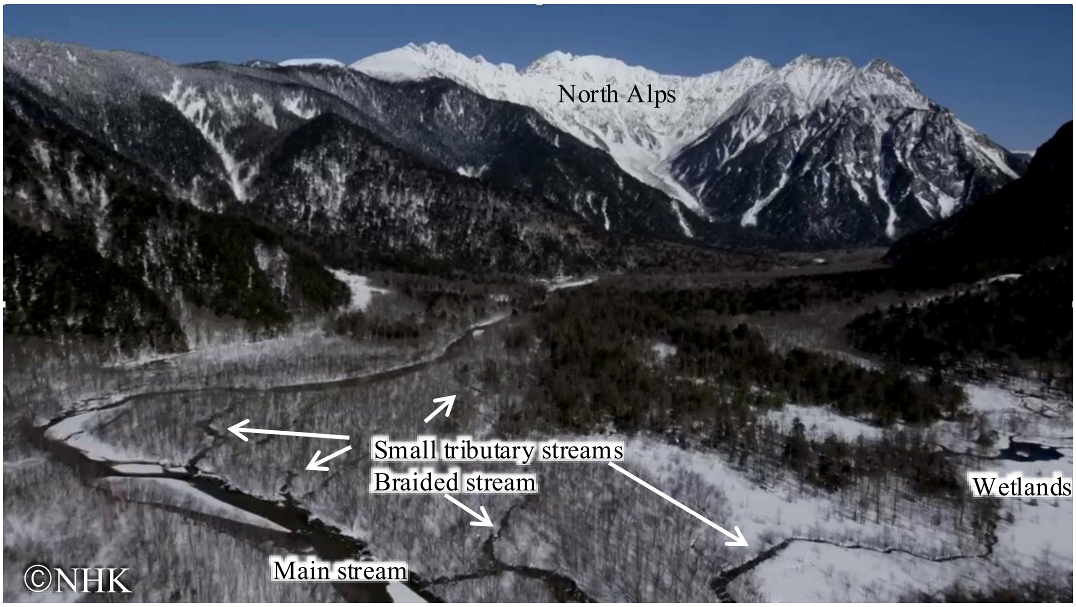

Figure S2 Study site and ranges of three Japanese macaque troops (KT, KK, KM troops) inhabiting Kamikochi targeted in this study. These ranges were estimated based on the locations recorded on the day before fecal samples were collected. Note that these ranges may be underestimated compared to their actual ranges.

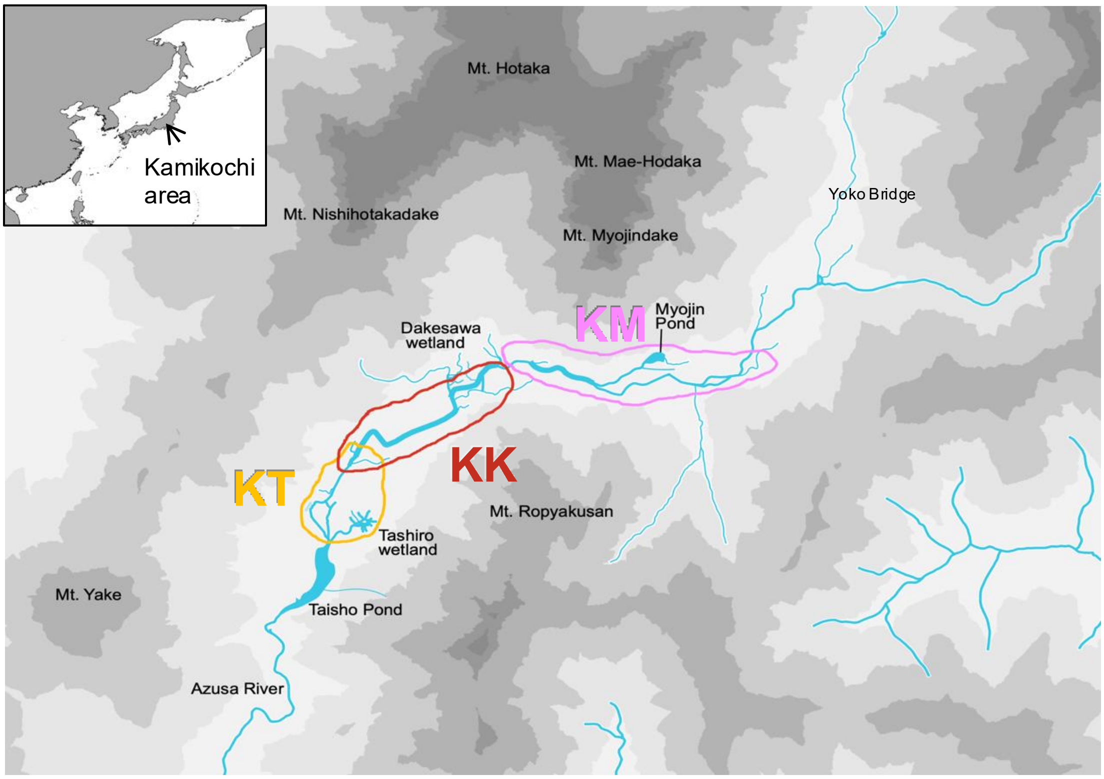

Figure S3 The study site and ranges of three Japanese macaque troops (KT, KK, KM troops) inhabiting Kamikochi targeted in this study. These ranges were estimated based on the locations recorded on the day before fecal samples were collected. Note that these ranges may be underestimated compared to their actual ranges. The map was created using the source program QGIS version 1.8 (https://qgis.org).

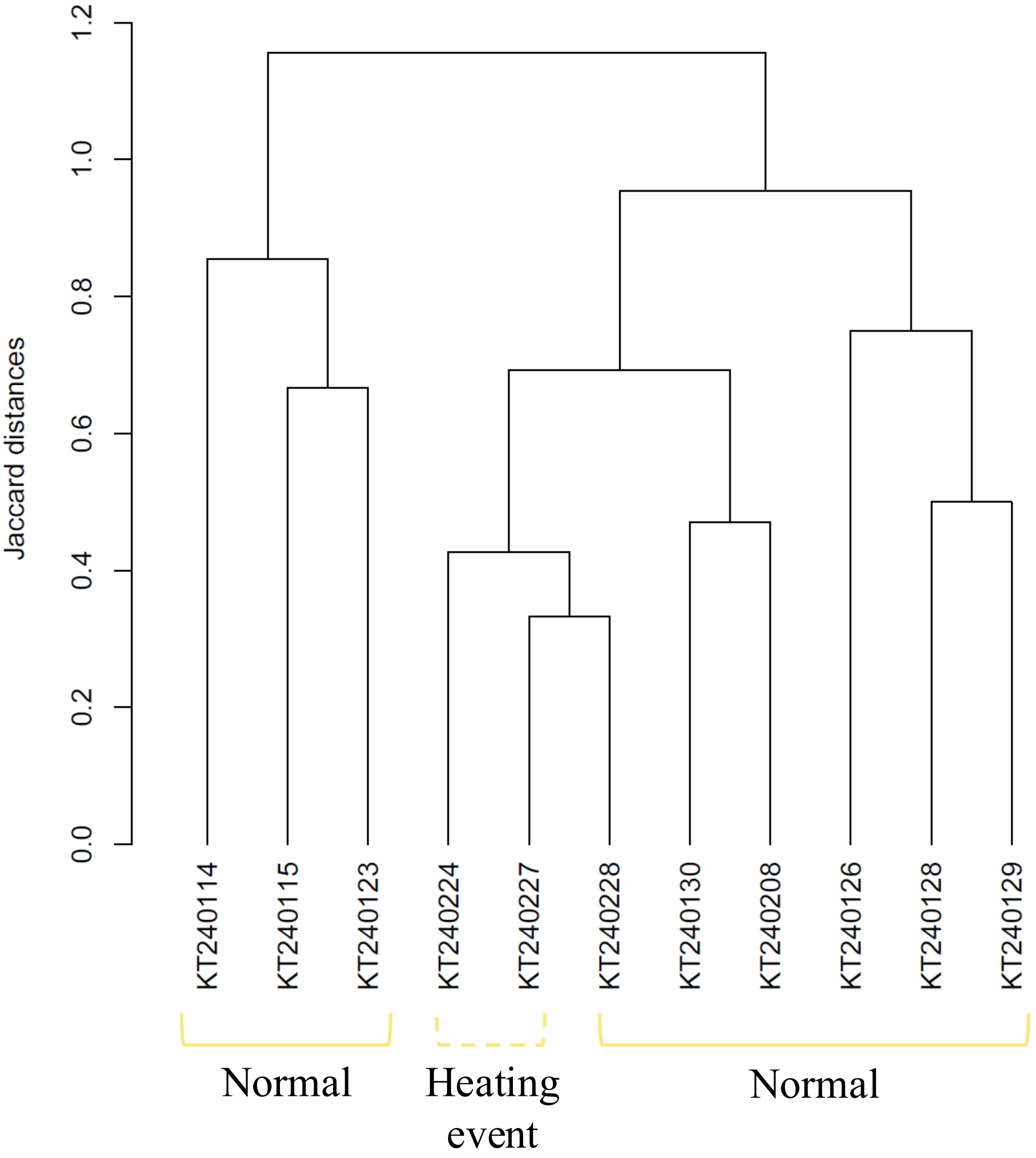

Figure S4 The community structure of insect species detected by DNA metabarcoding using the feces of the KT troop of Kamikochi macaques, by day, is shown for before and after the heating event. Differences in the community structure were detected between before and after the event.

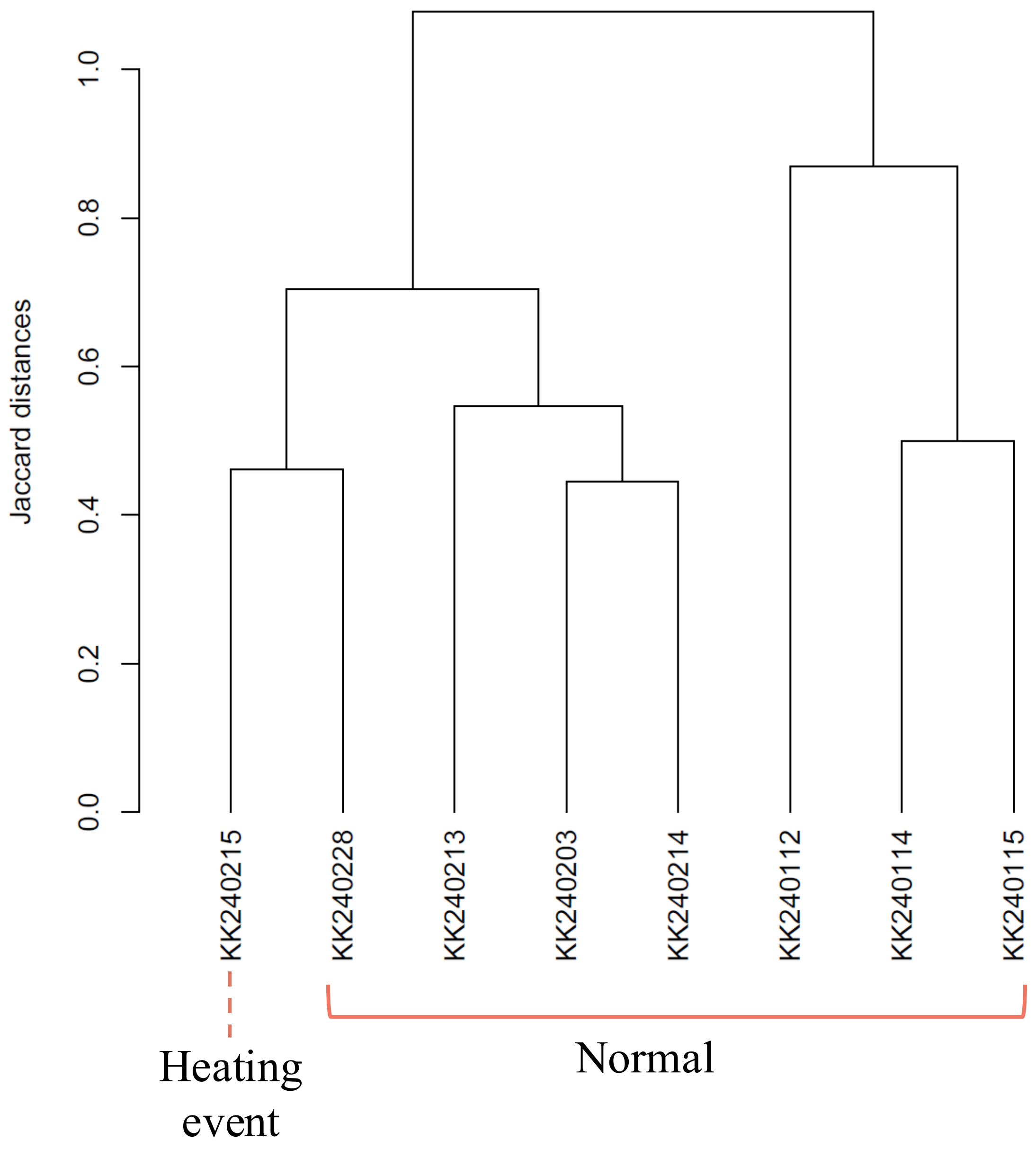

Figure S5 The community structure of insect species detected by DNA metabarcoding using the feces of the KK troop of Kamikochi macaques, by day, is shown for before and after the heating event. Differences in the community structure were detected between before and after the event.

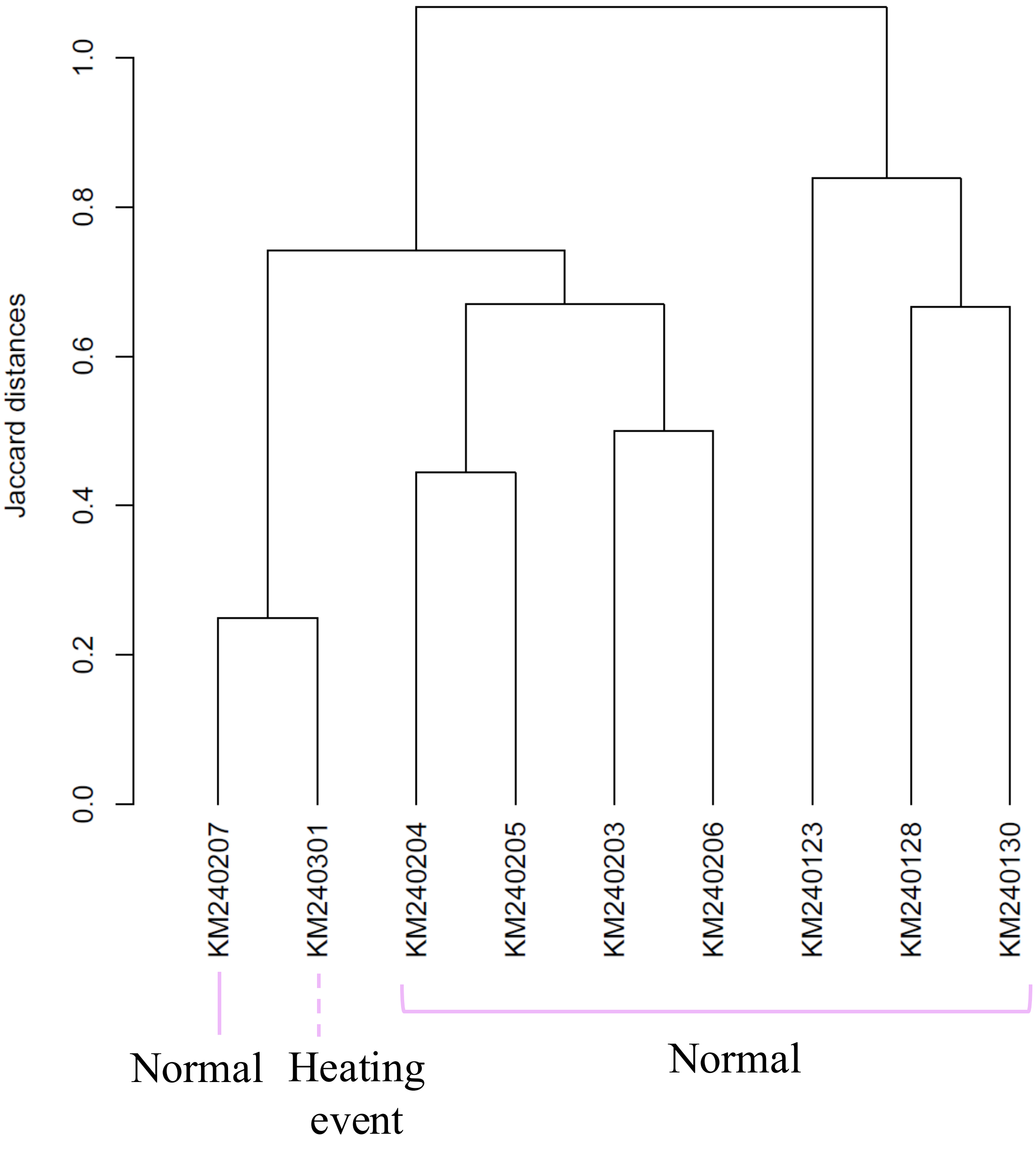

Figure S6 The community structure of insect species detected by DNA metabarcoding using the feces of the KM troop of Kamikochi macaques, by day. Days after the heating event were mostly not investigated.

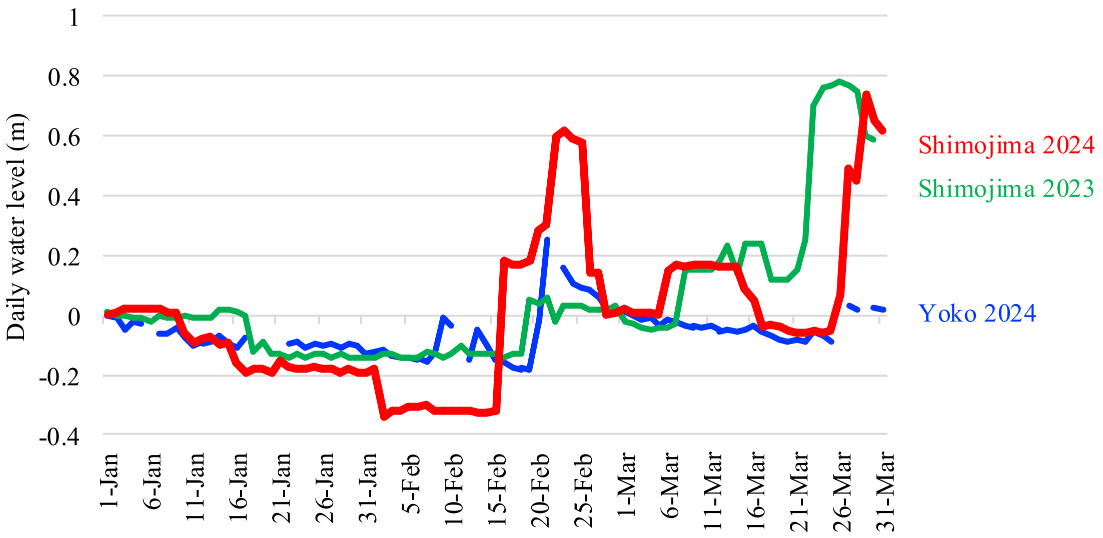

Figure S7. Daily changes in the river water level at Azusa River. The data of water level in Yoko Bridge in 2023 was excluded due to many missing data.

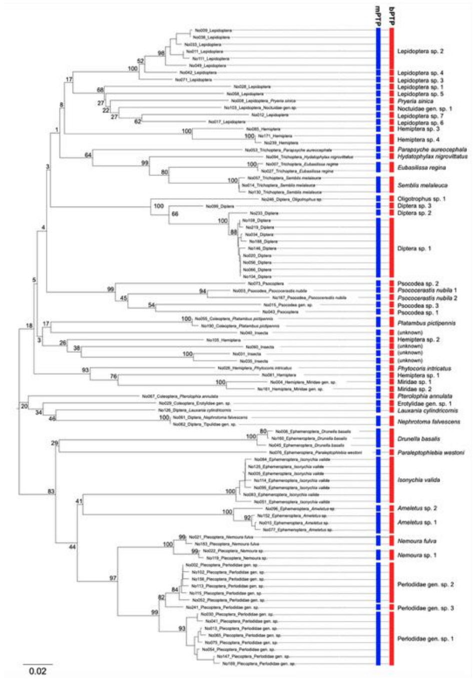

Figure S8 Phylogenetic tree by NJ methods based on the mtDNA 16S rRNA region. The numbers at the nodes represent the bootstrap values based on 1000 replications. The bPTP and mPTP analyses were conducted to estimate genetic species, and the results are shown to the right of the OTU. The results of both analyses were the same.
