# Supplementary material for: Impact of climate warming on the foraging behavior of northernmost distributed primates
Source: Sci Rep. 2025 Jul 25;15:27096. doi: 10.1038/s41598-025-09308-0 (PMC12297154; doi:10.1038/s41598-025-09308-0)
Supplement: Supplementary file 7 — Supplementary Information. [file 41598_2025_9308_MOESM7_ESM.docx]

**Impact of Climate Warming on the Foraging Behavior of Primates: Changes and Future Predictions**

Ema NAGAHARA^1,+^, Ayaka TSUCHIHASHI ^2^, Takumi YOSHIDA^3^, Kosuke HAYASHI^4^, Genki YAMADA^5^, Takayuki OGURA^6^, Mone ITO^6^, Hirokazu Kurihara^7^, Koji TOJO^1,8^, Takuya MATSUMOTO^1,8,*^, Masaki TAKENAKA^1,8,+,*^

^1^ Department of Biology, Faculty of Science, Shinshu University, 390-8621 Asahi 3-1-1, Matsumoto, Japan

^2^ Graduate School of Science and Technology, Shinshu University, 390-8621 Asahi 3-1-1, Matsumoto, Japan

^3^ Graduate School of Medicine, Science and Technology, Shinshu University, 390-8621 Asahi 3-1-1, Matsumoto, Japan

^4^ NHK Enterprises, Inc., 150-0047 Kamiyama 4-14, Shibuya, Tokyo, Japan

^5^ G-vision, Inc., 182-0006 Nishitsutsujigaoka 1-54-12, Chofu, Tokyo, Japan

^6^ Kozo Production, 150-0042 Udagawa 37-10-301, Shibuya, Tokyo, Japan

^7^Vision1 lnc., 150-0047 Shimochiai 3-15-20, Shinjyuku, Tokyo, Japan

^8^ Institute of Mountain Science, Shinshu University, 390-8621 Asahi 3-1-1, Matsumoto, Nagano, Japan

^+^ Co-First Author

^*^Co-Correspondence: Masaki Takenaka (masakiplayer@gmail.com) and Takuya Matsumoto ([matsumoto_t@shinshu-u.ac.jp](mailto:matsumoto_t@shinshu-u.ac.jp))

ORCID: Nagahara E, 0009-0009-3509-7117; Tsuchihasi A, 0009-0008-2068-897X; Yoshida T, 0009-0002-5743-3002; Tojo K, 0000-0002-9362-604X; Matsumoto T, 0000-0003-1363-9455; Takenaka M, 0000-0002-9565-524X

**Supplementary Information**

**Supplementary Information 1** Video of the foraging behavior of Japanese macaques: turning over stones in a stream and pinching aquatic insects with their fingers in Kamikochi.

**Supplementary Information 2** Video of the foraging behavior of Japanese macaques: turning over stones in a stream in which aquatic insects that had fallen off the stones and were carried away by the water flow were scooped up.

**Supplementary Information 3** Video of the foraging behavior of Japanese macaques: Kamikochi macaques sucking out tube case-making aquatic insects.

**Supplementary Information 4** Video of the foraging behavior of Japanese macaques: for nests that are hard and cannot be peeled off from rocks with their fingers, such as those of Trichoptera, they forage directly with their mouths.

**Supplementary Information 5** Video of Japanese macaques foraging on aquatic plants and algae in wetlands (with algae being the main focus in the video).

**Supplementary Information 6**

The age class of the Japanese macaques was determined as follows: individuals with exposed sex skins without hair were classified as adults, individuals with similar body size to adults, but with hair remaining on their sex skins, were classified as “adolescents”, individuals with relatively light body hair and particularly small bodies were judged to be in their first year and were classified as “infants”, individuals smaller than adolescents, but bigger than infants and judged to be 1-3 years old based on their body size, were classified as “juveniles”, and the rest were classified as “unknown”. Regarding females, individuals with confirmed nipple elongation due to breast-feeding were also classified as adults, even if it was not possible to confirm their sexual skin.

**Supplementary Information 7**

The foraging methods were categorized as follows: "pinch" by fingers, "scoop up" in the hands, "pinch" by fingers and then "suck out" from the insect case with their mouths (classified as "suck out"), and "forage directly with mouth". The insect species were identified as far as possible based on the videos, and the species were categorized into groups such as Crawlers, Swimmers, Net-spinners and Tube case-making groups to analyse their life forms. In this analysis, all behaviors were recorded as a single action, from capturing aquatic insects to bringing them to the mouth.

**Supplementary Information 8**

**DNA analysis**

For DNA extraction, small amounts (ca. 10 g) were sampled from the center of the feces and extracted using a QIAamp Fast DNA Stool Mini Kit (Qiagen). The extracted DNA was combined by day by troop. DNA from feces of the same troop on the same day was measured for concentration using a DS-11 Microvolume Spectrophotometer (DeNovix Inc., DE, USA), and adjusted to the same concentration. The combined DNA were sent to Bioengineering Lab. Co., Ltd. For PCR, the primers MtInsects-16S_F: GGACGAGAAGACCCTWTAGA and MtInsects-16S_R: ATCCAACATCGAGGTCGCAA^38^ were used. Since the feces contained a high amount of Japanese macaque DNA, a blocking primer (Macaca_16sF_Bloking_1F: gac cct atg gag ctt taa tct att aat gc -/3SpC3/) was used to inhibit the amplification of macaque DNA. S Sequencing of the DNA library was conducted as follows: The first PCR was performed using ExTaq HS (TaKaRa, Shiga, Japan) with primers and adapter sequences (F: 5' – ACA CTC TTT CCC TAC ACG ACG CTC TTC CGA TCT – 3' and R: 5' – GTG ACT GGA GTT CAG ACG TGT GCT CTT CCG ATC T). The first PCRs were conducted in 10 µL volumes (0.075 µL of ExTaq HS, 1.0 µL of 10xEx Buffer, 0.8 µL of dNTPs (each 2.5 mM), and 2 µL of DNA template). The following PCR protocol was used: 94℃ for 2min, 35× (94℃ for 30 sec, 54℃ for 15 sec, 50℃ for 30 sec, 72℃ for 30 sec), 72℃ for 5min. Then, VAHTS DNA Clean Beads (Vazyme) were added at a 1.0× volume of the PCR reaction solution, and the PCR products were purified. The second PCR was conducted using KOD FX Neo (TOYOBO, Osaka, Japan) to assign sequences an index to identify individual samples and bind them to the flow cell of a high-throughput sequencer. The second PCRs were conducted in 10 µL volumes (0.2 µL of KOD FX Neo (1.0 U/uL), 5.0 µL of 2× PCR Buffer for KOD FX Neo,2.0 µL of dNTPs (each 2.5 mM), and 1.0 µL of PCR product). The following PCR protocol was used: 94℃ for 2min, 35× (98℃ for 10 sec, 60℃ for 30 sec, 68℃ for 30 sec), 68℃ for 2min. Then, VAHTS DNA Clean Beads (Vazyme) were added at a 1.0× volume of the PCR reaction solution, and the PCR products were purified. The sequence of the library prepared was performed using the MiSeq platform (Illumina, San Diego, CA, USA) of the Reagent Kit v3 (2x300 bp). All raw sequencing reads were registered with the DNA Data Bank of Japan (DDBJ: LC859418-LC859545).

**Data analysis**

Read quality control and filtering were performed using fastp (Chen et al., 2018) with the following parameters: -q 30 (quality score ≥30), -u 40 (removal of reads with more than 40% low-quality bases), and -l 50 (removal of reads shorter than 50 bp). Primers were removed using the cutadapt plugin in QIIME2^40^ with the --p-discard-untrimmed option enabled, which excluded sequences for which primer sequences were not detected. Then, denoising and error correction were performed using DADA2^41^. In this study, the mtDNA 16S rRNA region was used, and since the sequence lengths vary by species, no sequence length restrictions were applied, following DADA2 ITS Pipeline Workflow (1.8) (<https://benjjneb.github.io/dada2/ITS_workflow.html>) as a reference. Sequences with fewer than four reads were removed using the filter-feature function. The obtained sequences were subjected to BLAST searches using local BLAST 2.13.0+ (<https://ftp.ncbi.nlm.nih.gov/blast/executables/blast+/LATEST/>). BLAST searches were conducted using a reference database comprising three sources: (1) Due to the lack of a comprehensive DNA database for insects, we analyzed the target DNA barcoding region using previously collected samples in this study and registered the sequences in DDBJ (accession number: LC859418-LC859545). Sequences from the mtDNA 16S rRNA DNA barcoding region previously collected from insect samples in Kamikochi., (2) Sequences from GenBank (https://blast.ncbi.nlm.nih.gov/Blast.cgi) with the query "Animalia 16S NOT 'whole genome' NOT 'chromosome' NOT 'complete genome'," resulting in 423,750 (May 27, 2024), and (3) The insect DNA database created by Kanagawa Prefecture (<https://www.pref.kanagawa.jp/docs/b4f/suigen/edna-en.html>). For sequences obtained from the BLAST search, the one with the highest sequence identity was selected. The threshold for the BLAST search was set at 1e-40, and taxonomic identification was performed based on sequence identity: ≥98% for species level, ≥97% for genus level, ≥94% for family level, and ≥85% for order level.

**References**

38. Takenaka, M., Yano, K., Suzuki, T., & Tojo, K. Development of novel PCR primer sets for DNA barcoding of aquatic insects, and the discovery of some cryptic species. *Limnology* **24**, 121–136 (2023). <https://doi.org/10.1007/s10201-022-00710-5>

40. Bolyen, E. et al. Reproducible, interactive, scalable and extensible microbiome data science using QIIME 2. *Nat. Biotechnol.* **37**, 852-857 (2019). <https://doi.org/10.1038/s41587-019-0209-9>

41. Callahan, B. J., McMurdie, P. J., Rosen, M. J., Han, A. W., Johnson, A. J. A., & Holmes, S. P. DADA2: High-resolution sample inference from Illumina amplicon data. Nat. Methods **13**, 581-583 (2016). <https://doi.org/10.1038/nmeth.3869>

**Supplementary Information 9**

The BLAST results rely on species identification based on sequence similarity, which is not highly accurate. Additionally, the number of species registered in the database is limited, restricting the range of species that can be identified. Therefore, to conduct species identification for samples identified at the family or genus level, we incorporated interpretations based on phylogenetic analysis. About a species delimitation analysis, the OUTs identified as Insecta through BLAST search were subjected to NJ tree construction (1000 bootstrap) using MEGA ver. 7.0.26^39^. Sequence alignment was performed using the MAFFT server (<https://mafft.cbrc.jp/alignment/server/>)^42^. The tree file was exported in newick format, and genetic species estimation was conducted using the bPTP web server^43^ (<https://species.h-its.org/>), the mPTP web server^44^ (<https://mptp.h-its.org/#/tree>). In the analysis, the PTP model was selected for the mPTP analysis, and all other settings were run with default values (Fig. S8). The results of the bPTP analysis were used to complement information for those species that could not be identified at the species level by BLAST, supplementing the genetic species delineation.

**References**

39. Kumar, S., Stecher, G., & Tamura, K. MEGA7: molecular evolutionary genetics analysis version 7.0 for bigger datasets. *Mol. Biol. Evol.* **33**, 1870-1874 (2016). <https://doi.org/10.1093/molbev/msw054>

42. Katoh, K., & Standley, D. M. MAFFT Multiple sequence alignment software version 7: Improvements in performance and usability. *Mol. Biol. Evol.* **30**, 772–780 (2013). <https://doi.org/10.1093/molbev/mst010>

43. Zhang, J. et al. A general species delimitation method with applications to phylogenetic placements. *Bioinformatics* **29** 2869-2876 (2013). <https://doi.org/10.1093/bioinformatics/btt499>

44. Kapli, P. et al. Multi-rate Poisson tree processes for single-locus species delimitation under maximum likelihood and Markov chain Monte Carlo. *Bioinformatics* **33**, 1630-1638 (2017). <https://doi.org/10.1093/bioinformatics/btx025>

**Supplemental Figure Legends**

**Figure S1** Mean winter temperatures at various locations on the Japanese Islands where inhabit macaques, including Yakushima (southernmost area), Kyoto, Kamikochi (the study area), Kinkasan (island in northeastern Japanese Islands), and the Shimokita Peninsula (northernmost area) (Dec 1, 2023 – Mar 31 2024). The temperature in Kamikochi is lower than in any other region where macaques are distributed. In particular, it is colder than the Shimokita Peninsula, which is the northernmost distribution range of non-human primates. The temperature and precipitation data for this study were obtained using observation data from the Research Center for Mountain Environment, Shinshu University (http://ims.shinshu-u.ac.jp/~metims_web/index.php?sokuhou) and from the Japan Meteorological Agency.

**Figure S2** Study site and ranges of three Japanese macaque troops (KT, KK, KM troops) inhabiting Kamikochi targeted in this study. These ranges were estimated based on the locations recorded on the day before fecal samples were collected. Note that these ranges may be underestimated compared to their actual ranges.

**Figure S3** Community structure of insect species detected by DNA metabarcoding using feces collected from the KT troop of Kamikochi macaques, by day, is shown for before and after the heating event. Differences in the community structure were detected between before and after the event.

**Figure S4** Community structure of insect species detected by DNA metabarcoding using feces collected from the KK troop of Kamikochi macaques, by day, is shown for before and after the heating event. Differences in the community structure were detected between before and after the event.

**Figure S5** Community structure of insect species detected by DNA metabarcoding using feces collected from the KM troop of Kamikochi macaques, by day. Days after the heating event were mostly not investigated.

**Figure S6** Daily changes in the stream water level at Azusa River. Data on the water level in Yoko Bridge in 2023 were excluded as they were incomplete.

**Figure S7** Phylogenetic tree by NJ methods based on the mtDNA 16S rRNA region. The numbers at the nodes represent the bootstrap values based on 1000 replications. The bPTP and mPTP analyses were conducted to estimate genetic species, and the results are shown to the right of the OTU. The results of both analyses were the same.
